# Fracture Limits of Maxillary Fourth Premolar Teeth in Domestic Dogs Under Applied Forces

**DOI:** 10.3389/fvets.2018.00339

**Published:** 2019-01-30

**Authors:** Maria Soltero-Rivera, Matthew I. Elliott, Michael W. Hast, Snehal S. Shetye, Ana C. Castejon-Gonzalez, Lenin A. Villamizar-Martinez, Darko Stefanovski, Alexander M. Reiter

**Affiliations:** ^1^Department of Clinical Sciences and Advanced Medicine, School of Veterinary Medicine, University of Pennsylvania, Philadelphia, PA, United States; ^2^Mars Petcare Care and Treats, Europe, Birstall, United Kingdom; ^3^The McKay Orthopedic Research Laboratory, University of Pennsylvania, Philadelphia, PA, United States; ^4^Department of Clinical Sciences, North Carolina State University College of Veterinary Medicine, Raleigh, NC, United States; ^5^Department of Clinical Studies-New Bolton Center, School of Veterinary Medicine, University of Pennsylvania, Kennett Square, PA, United States

**Keywords:** tooth fracture, small animal dentistry, veterinary, trauma, endodontic disease

## Abstract

A cadaveric study was performed to investigate the external mechanical forces required to fracture maxillary fourth premolar teeth in domestic dogs and describe a clinically relevant model of chewing forces placed on functionally important teeth in which fracture patterns are consistent with those defined by the American Veterinary Dental College (AVDC). Twenty-four maxillary fourth premolar teeth were harvested from dog cadavers. Samples consisted of teeth with surrounding alveolar bone potted in polycarbonate cylinders filled with acrylic. The cylinders were held by an aluminum device at an angle of 60° with respect to the ground. An axial compression test was performed, creating a force upon the occluso-palatal aspects of the main cusps of the crowns of the teeth. The highest compressive force prior to failure was considered the maximum force sustained by the teeth. Results showed the mean maximum force (± SD) sustained by the tested teeth at the point of fracture was 1,281 N (± 403 N) at a mean impact angle (± SD) of 59.7° (± 5.2°). The most common fracture type that occurred among all samples was a complicated crown fracture (*n* = 12), followed by an uncomplicated crown fracture (*n* = 6), complicated crown-root fracture (*n* = 5), and uncomplicated crown-root fracture (*n* = 1). There was no statistically significant correlation between dog breed, age, weight, impact angle, crown height or crown diameter, and the maximum force applied at the point of fracture. The only independent variable that remained significantly associated with maximum force was the crown height to diameter ratio (*p* = 0.005), suggesting that a decreased ratio increases tooth fracture resistance. The methodology described herein has been successful in creating a pattern of fracture of maxillary fourth premolar teeth consistent with that defined by the AVDC under angled compression at forces within the maximum chewing capability of the average domestic dog.

## Introduction

Tooth fracture in dogs is a commonly observed clinical condition, with a reported prevalence of 20–27%, although the literature quantifying this number is limited^1^ (1–6). In maxillofacial trauma patients the reported prevalence is higher (67–85%) (4, 7). Tooth fractures often occur as a result of traumatic impacts, such as road traffic accidents, but there is increasingly latent concern about the potential role of chewing on treats and toys in the fracture of large cheek teeth (8, 9). Tooth fractures have been reported to occur most commonly in functionally important teeth that play a role in prehension and chewing^1^ (1, 4–7).

Enamel of dogs has been reported to be thinner than that of humans, varying in thickness from 0.1 to 1 mm (10). In addition to a reduced thickness, other significant differences between human and carnivore enamel have been described in the inner layer, the configuration of Hunter-Schreger bands, and the content of the most superficial layer (11). The mechanical properties of human enamel and dentin have been well-defined (12), but they have been insufficiently investigated in dogs. The implication of substantially thinner enamel for tooth robustness has not been explored. Dentinal width of a vital tooth (i.e., one with living pulp tissue) is known to increase with age, with concentric thickening of secondary dentin occurring throughout most of the animal's life. Tertiary dentin can also be produced in response to an insult (13).

The role of chewing activity in the development of tooth fractures in dogs remains controversial, in part due to the lack of information available about the resilience of teeth to masticatory forces and the impact of the treat or toy's textural properties in transmitting a catastrophic stress concentration through the hard tissue layer of teeth. In addition to this, stress distribution on functionally important teeth during chewing activity has not been evaluated (14).

Mechanical testing conducted on at least one of the commonly offered hard treats and toys has shown material properties that surpass those reported for human enamel and dentin (15). Dogs can generate voluntary bite forces ranging from 13 to 1,394 N (16), which has been shown to be directly proportional to their size (17). When all four canine teeth are involved, the maximum pulling force can range from 480 to 1,200 N (18). However, caution must be exercised when attempting to quantify the extent to which animals voluntarily deploy chewing forces (19). It is known from modeling and stimulated chewing under anesthesia that maximum potential bite forces for dogs can be significantly higher, peaking at over 3,400 N at molar teeth (17).

The functionally important maxillary fourth premolar and mandibular first molar teeth (“carnassial teeth”) are not only commonly employed in chewing but also subject to higher forces than canine teeth due to lever effects within their jaws (17). Fractures of the maxillary fourth premolar teeth often are complicated crown or crown-root fractures, exposing the pulp, and causing discomfort to the animal (1–3, 20). Therefore, prevention of dental trauma requires an understanding of the process of tooth fracture and information on the textural quality of chewing materials. The fact that half of owners of dogs with fractured teeth do not notice tooth fractures further supports this need (20).

For the purpose of evaluating the potential effects of material texture on the risk of inducing a maxillary fourth premolar tooth fracture during chewing, it is necessary to combine the referenced knowledge for chewing forces with an understanding of the forces required to induce a clinically observed tooth fracture. Mechanical testing has previously been conducted on extracted canine teeth from dogs, using a universal materials testing machine after potting the teeth in acrylic and applying the force at a speed of 1 mm/min to the disto-occlusal line angle at an angle of 45° to the long axis of the crowns (21). The mean forces required to fracture canine teeth ranged from 494 to 630 N depending on the crown height to diameter ratio. A fracture force of approximately 890 N has previously been suggested for maxillary fourth premolar teeth, although detailed information about methodology and results were not provided for that study.^2^ Other studies investigated the fracture forces of canine teeth in dogs for pulling/tugging purposes with focus on service animals (18). One study explored the effect of dental restoration technologies on the subsequent fracture forces of teeth post restoration.^3^ A lower crown height to diameter ratio has been found to increase fracture resistance of canine teeth that have undergone endodontic and prosthodontic therapy (21). Further literature on this subject is sparse at best, and this in part explains the lack of established guidelines for recommended chewing textures of treats and toys.

The present study aimed to increase our knowledge about the fracture limits of maxillary fourth premolar teeth from dogs under chewing conditions by *in vitro* testing, replicating the crown and crown-root fractures observed clinically, and to use that new information for future studies that establish criteria for increased risk of tooth fracture with regards to the chewing textures of treats and toys. Another goal was to determine the impact of signalment of the dog (breed, age, and weight) and physical characteristics of the tooth (crown height, crown diameter, and crown height to diameter ratio) on the force needed to cause tooth fracture.

## Materials and Methods

### Cadaveric Harvesting

Cadaver heads of domestic dogs euthanized for causes unrelated to this study were assessed.^4^ Age of the dogs was known or estimated based on root canal width, evidence of coronal wear, severity of periodontal disease, and calculus accumulation. Weights of the dogs were unknown and estimated by predominant breed and head size and categorized as: < 10 kg, 10–14, 15–19, 20–24, 25–29, 30–34 kg, and >34 kg. Inclusion criteria were, except for mild coronal wear, otherwise structurally intact maxillary fourth premolar teeth without prior endodontic, restorative or prosthodontic treatment and without radiographic signs of disease.

Cadaver heads were refrigerated at 35°F (1.67°C) until sample harvesting. The maxillary fourth premolar teeth were collected by making osteotomies through the dorsolateral and palatal aspects of the maxilla as well as the distal aspect of the third premolar and mesial aspect of the first molar teeth (to avoid injury to the subject teeth) following soft tissue reflection. The bone was initially scored using a water-cooled #701, cross-cut, fissure, carbide bur attached to a high-speed dental handpiece. Remaining bony attachments were cut with an osteotome and mallet.

**Figure 1:**
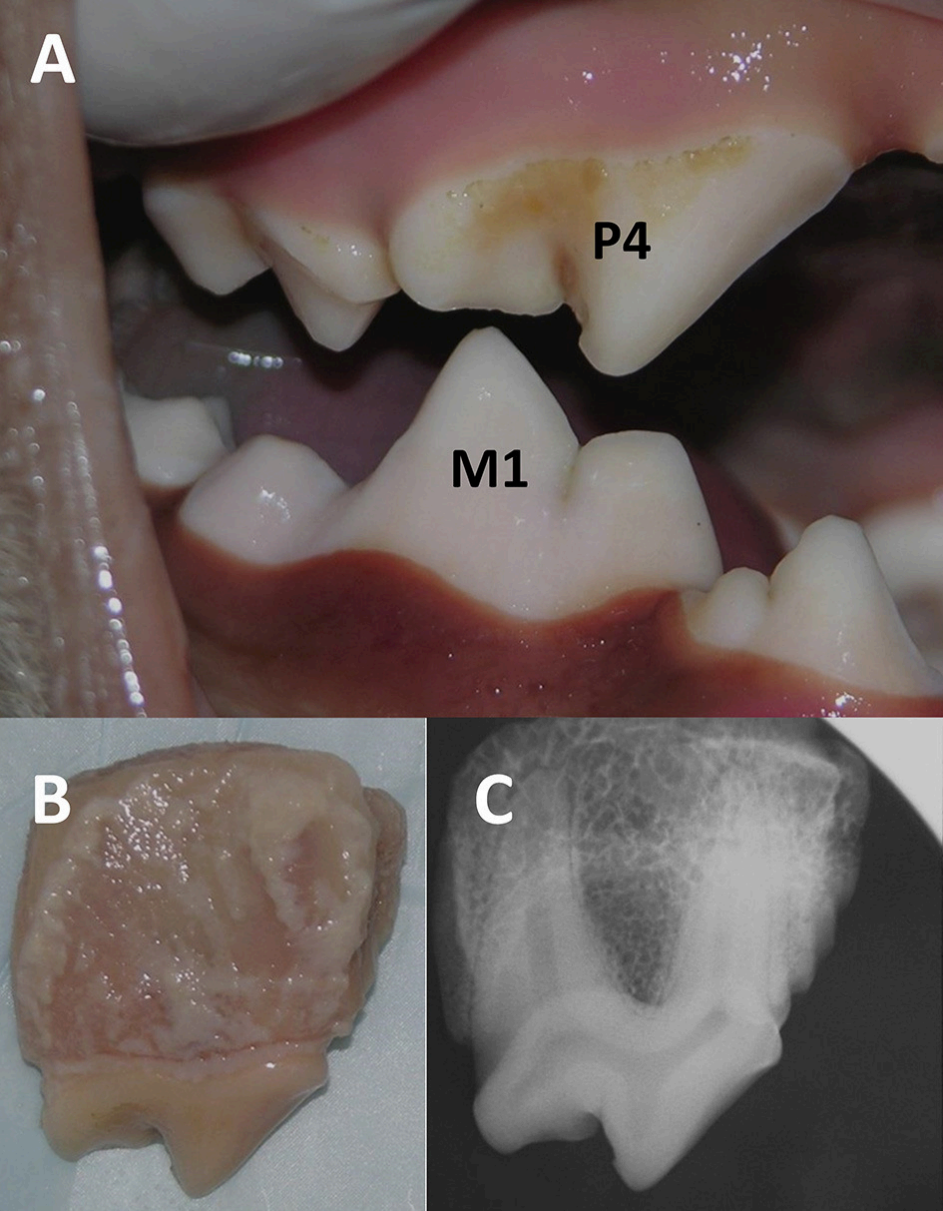
Photograph showing occlusal relationship between the right maxillary (i.e., fourth premolar, P4) and mandibular (i.e., first molar, M1) carnassial teeth in a domestic dog cadaver **(A)**. The harvested right maxillary fourth premolar tooth with surrounding bone was photographed **(B)** and radiographed **(C)**.

Loose alveolar mucosa and gingiva, and in some cases also remnants of adjacent teeth still attached to the samples, were removed. The harvested maxillary fourth premolar teeth surrounded by alveolar bone were photographed and radiographed to confirm teeth included in the sample were harvested in their entirety (Figure 1) before being placed in lactated Ringer's solution and stored at 24.8°F (−4°C). The crown height was measured from the furcation between the mesiobuccal and distal roots to the most coronal point of the crown. The crown diameter was measured from the most mesial to the most distal aspect of the crown.

### Biomechanical Testing

Prior to conducting the present study, preliminary testing was performed with individual teeth potted in PMMA vs. teeth with surrounding gingiva and bone being potted in PMMA as well as with utilizing different impact angles. Teeth with surrounding gingiva and bone allowed for easier and more repeatable potting, appeared more realistic, and when being subjected to a vector of force at impact angles of about 50 to 70° resulted in fracture patterns that are consistent with those defined by the American Veterinary Dental College (AVDC).

The maxillary fourth premolar teeth surrounded by alveolar bone were left to defrost at room temperature before being potted in 57.15 mm outer diameter polycarbonate cylinders filled with poly(methyl-methacrylate) (PMMA).^5^ Each sample was seated into the acrylic close to the cementoenamel junction and oriented as perpendicular as possible to the flat bottom surface of the pot. While the PMMA was allowed to cure for 1 h, the samples were covered with gauze slightly damped with phosphate buffered solution (PBS). Once the PMMA had cured, the samples were stored in containers of PBS at 39.2°F (4°C) until the time of testing.

**Figure 2:**
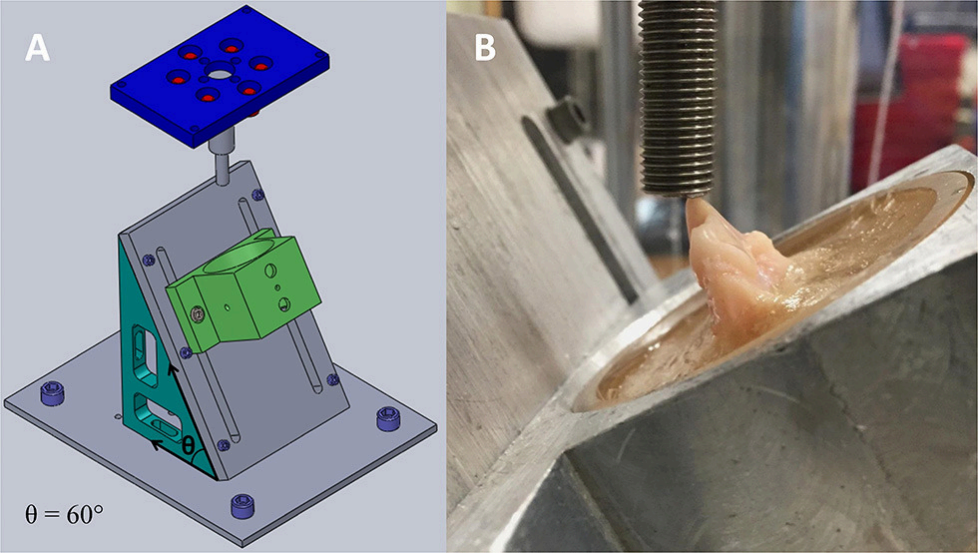
Computer-generated drawing that depicts the aluminum jig used to create compressive loading in a controlled manner. The device was holding each pot at an angle of 60° (θ) with respect to the ground **(A)**. Right maxillary fourth premolar tooth with surrounding alveolar bone seated inside a polycarbonate cylinder filled with PMMA. The pot is held by the aluminum device, and the steel actuator is coming into contact with the occluso-palatal aspect of the main cusp of the tooth at the onset of mechanical testing **(B)**.

The PMMA-filled polycarbonate cylinders were securely fixed to the aluminum frame. The samples were randomized and underwent an axial compression test on a servo-hydraulic universal testing machine equipped with a 10 kN/50 Nm load/torque cell.^6^ A 9.40 mm diameter stainless steel threaded rod was used in conjunction with the actuator to create a compressive force upon the crowns of the teeth. In order to simulate the forces generated by the occlusion of the maxillary fourth premolar teeth against a treat or toy, a custom-built aluminum device was used to hold each pot at an angle of 60° with respect to the ground (Figure 2). The occluso-palatal aspect of the main cusp of the crown of the tooth was facing the incoming onset of compression to create a point of contact with the actuator. Photographs were taken of each sample prior to testing, and the exact impact angle of the tooth with respect to the actuator was calculated using Image J^7^ in order to determine the exact angulation between the face of the actuator and the long axis of the tooth.

**Figure 3:**
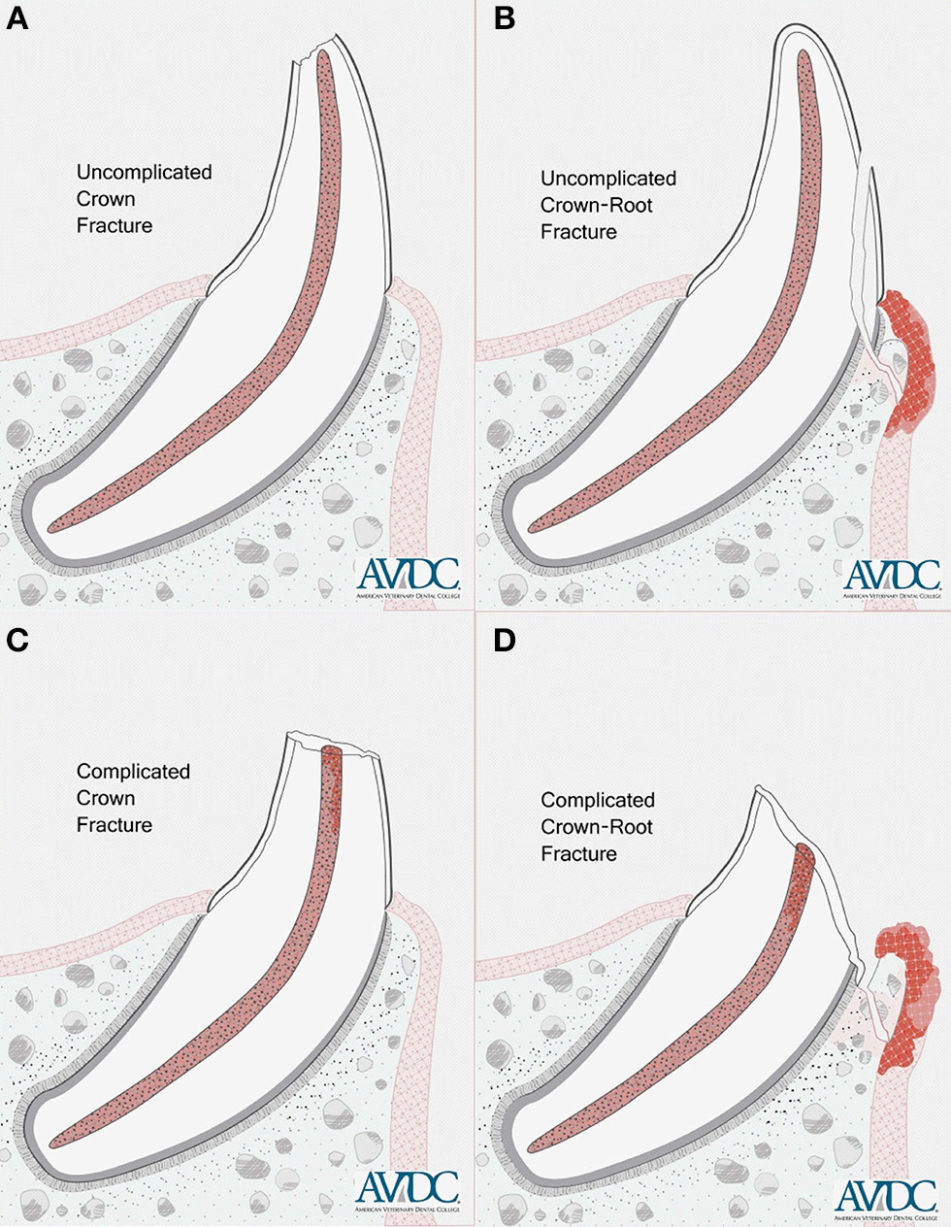
Illustrations showing an uncomplicated crown fracture **(A)**, uncomplicated crown-root fracture **(B)**, complicated crown fracture **(C)** and complicated crown-root fracture **(D)** of a single-rooted tooth. Copyright American Veterinary Dental College (AVDC), used with permission.

All samples were subjected to a 10 N preload and subsequently tested to failure at a rate of 0.1 mm/s. Failure was classified as an instantaneous decrease of force greater than or equal to 50%. The highest force prior to failure was considered the maximum force sustained by the tooth. Samples were analyzed photographically, and fracture types were determined based on a classification of the AVDC,^8^ which considers an uncomplicated crown fracture to be a fracture of the crown that does not expose the pulp, a complicated crown fracture a fracture of the crown that exposes the pulp, an uncomplicated crown-root fracture a fracture of the crown and root that does not expose the pulp, and a complicated crown-root fracture a fracture of the crown and root that exposes the pulp (Figure 3).

### Statistical Analysis

All statistical analysis was performed using JMP 13 (SAS, Cary, NC, USA) and STATA 15.1 MP (StataCorp, College Station TX). A *p* < 0.05 was considered to be statistically significant. For continuous variables, descriptive statistics were reported as means and standard deviation (mean ± SD). Frequency counts and percentages were used for reporting categorical variables (dog breed, weight, and others). Exploratory statistical analysis was performed using univariate linear regression with the maximum force as the dependent (outcome) variable. Independent variables that were tested for association with the main outcome included dog breed, age, weight, impact angle, fracture type, crown height, crown diameter, and crown height to diameter ratio. All variables that showed statistical trends (*p* < 0.3) for association with main outcome were retained for further statistical analysis. Backwards stepwise selection (iterative F-tests) was applied to investigate the interactions between these variables.

## Results

**Figure 4:**
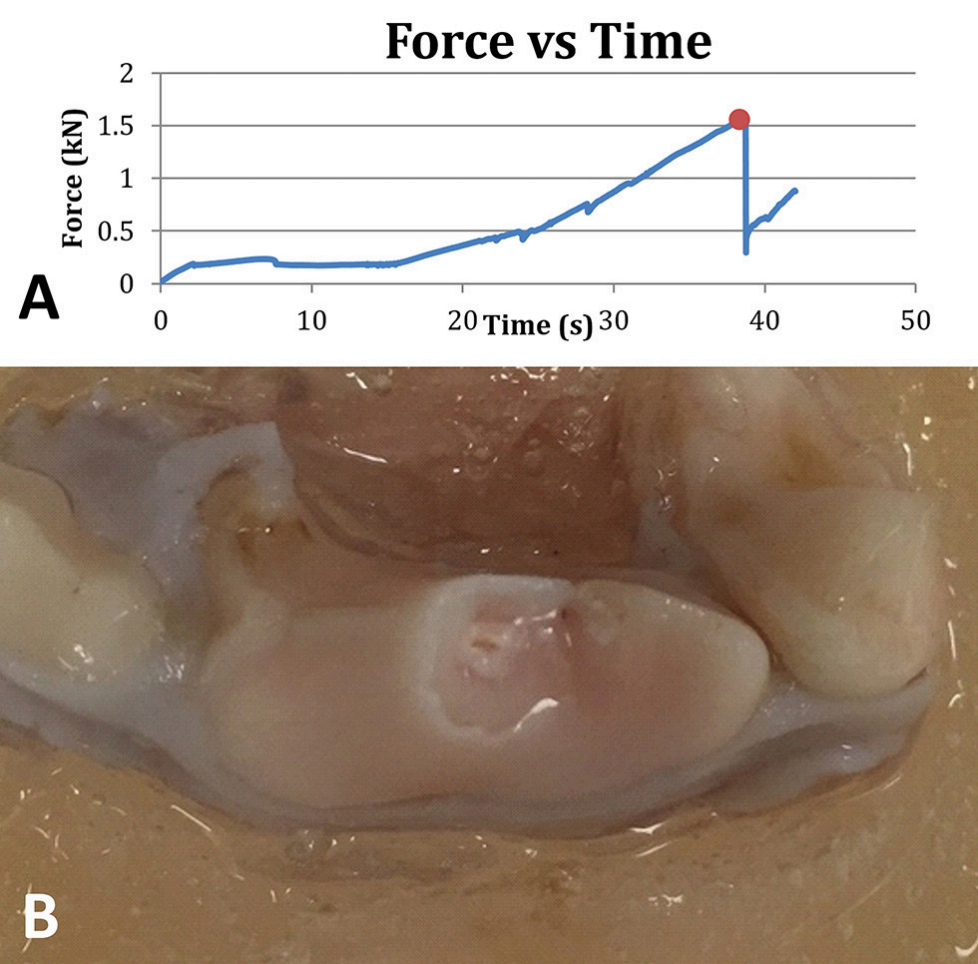
A representative plot of the force applied to a tooth as a function of time. The ultimate force before instantaneous failure is denoted with a red dot **(A)**. Right maxillary fourth premolar tooth showing a complicated crown fracture (i.e., with pulp exposure) following mechanical testing **(B)**.

**Figure 5:**
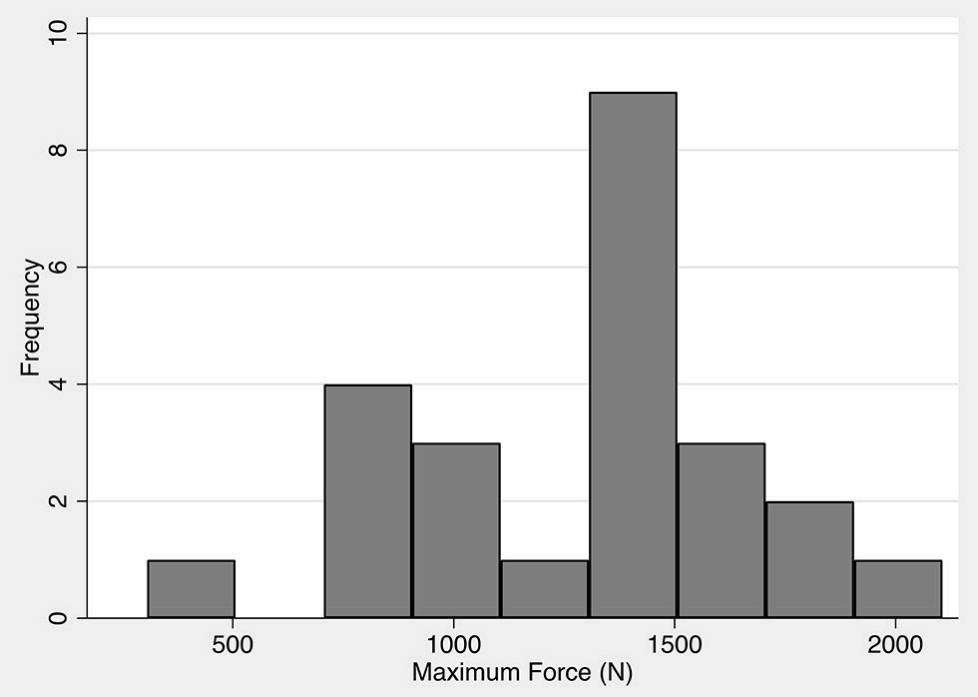
Distribution of forces leading to fracture of 24 maxillary fourth premolar teeth harvested from domestic dog cadavers. The maximum force is normally distributed with a mean force of 1,281 N and a standard deviation of 403 N.

Results from 24 compressive load tests were pooled for analysis. The mean maximum force (± SD) sustained by the tested teeth prior to fracture was 1,281 N (± 403 N) at a mean impact angle (± SD) of 59.7° (± 5.2°). Load-displacement plots from mechanical testing showed minor fracture events evidenced by small drops in force prior to catastrophic failure, but these were not considered fractures that compromised the mechanical integrity of the tooth (Figure 4). The most common fracture type that occurred among all samples was a complicated crown fracture (*n* = 12). An uncomplicated crown fracture was found in six samples, an complicated crown-root fracture in five samples, and an uncomplicated crown-root fracture in one sample. A summary of demographic and mechanical testing data can be found in Table 1. The distribution of the force to failure for all the samples is shown in Figure 5.

**Table 1:** Summary of data obtained from 24 maxillary fourth premolar teeth of domestic dogs with known or estimated breed, weight and age including mechanical testing performance in terms of maximum force until fracture at a calculated impact angle and resulting fracture type.

**Sample**	**Breed**	**Weight (kilograms)**	**Age (years)**	**Maximum force (N)**	**Impact angle (^**°**^)**	**Fracture type**	**Crown height (mm)**	**Crown diameter (mm)**	**Crown height to diameter ratio**
1	Mixed	< 10	3	916	61.1	UCF	15	11	0.733
2	Mixed	< 10	3	1,334	60.3	UCF	16	11	0.688
3	Beagle	10–14	4	735	62	UCF	16	10	0.625
4	Beagle	10–14	4	1,027	59.1	CCRF	16	10	0.625
5	Beagle	10–14	4	861	58.6	CCF	16	11	0.688
6	Mixed	30–34	8	1,387	62.4	CCF	20	12.5	0.625
7	Mixed	30–34	8	1,349	58	CCF	20	12.5	0.625
8	Staffordshire terrier	25–29	3	1,376	57.4	CCF	21	12	0.571
9	Staffordshire terrier	25–29	3	1,829	67.1	UCRF	21	12.5	0.595
10	German shepherd mix	>34	3	1,312	58.9	CCF	21	11.5	0.548
11	German shepherd mix	>34	3	2,029	58.8	CCRF	21	11.5	0.548
12	Staffordshire terrier mix	>34	1.5	1,682	61.7	CCF	20	10	0.5
13	Beagle	10–14	6	1,553	52.4	CCF	18	11	0.611
14	Beagle	10–14	3	1,454	69.3	CCF	16	10.5	0.656
15	Brittany spaniel	15–19	3	1,422	59.2	CCF	18	11	0.611
16	Hound mix	15–19	3	1,005	64.8	CCF	19	11	0.579
17	Hound mix	10–14	6	1,312	54.4	CCF	18	10.5	0.583
18	Beagle	10–14	7	1,322	62.8	CCF	17	11	0.647
19	Mixed	>34	10	1,889	48.4	CCRF	21	12	0.571
20	Bernese mountain dog	>34	3	306	47.3	CCRF	20	12	0.6
21	Mixed	20–24	3	1,650	61	CCRF	21	12.5	0.595
22	Mixed	15–19	1.5	820	60.7	UCF	18	11	0.611
23	Mixed	20–24	2.5	900	68.1	UCF	22	13	0.591
24	Mixed	15–19	2	1,283	58.4	UCF	19	11	0.579

*N, Newton; °, Degree;mm, Millimeter; UCF, Uncomplicated crown fracture; CCF, Complicated crown fracture; UCRF, Uncomplicated crown-root fracture; CCRF, Complicated crown-root fracture*.

**Figure 6:**
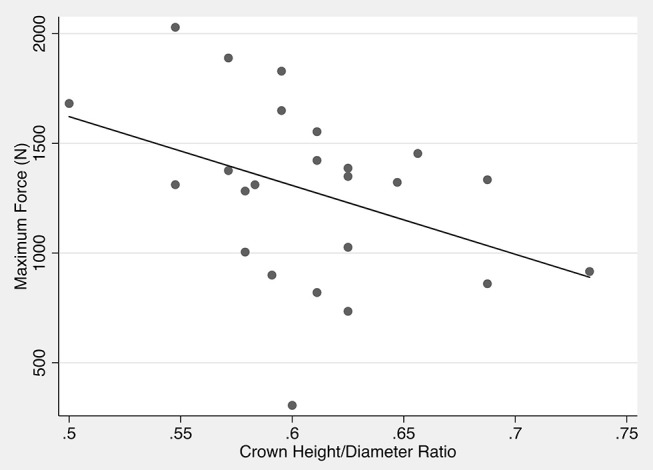
Scatter plot of crown height to diameter ratio vs. maximum force. The line indicates the predicted maximum force based on the crown height to diameter ratio.

Univariate exploratory statistical analysis revealed no statistically significant correlation between maximum force and impact angle (*p* = 0.841) for all teeth evaluated in the study. Crown height also showed no trend for being associated with maximum force (*p* = 0.4601). However, maximum force showed trends for association when compared to dog age (*p* = 0.124) and weight (*p* = 0.243), fracture type (*p* = 0.206), and crown diameter (*p* = 0.055). Furthermore, the crown height to diameter ratio showed significant association with maximum force (*p* = 0.005). Multiple linear regression model was fit against the maximum force using variables that showed significant trends of association with the outcome. Backwards stepwise regression was used with a goal to minimize the Akaike information criterion (AIC) to identify significant effects. The only independent variable that remained significantly associated with maximum force was the crown height to diameter ratio (*p* = 0.005) (Figure 6). For every one unit increase in the crown height to diameter ratio, the maximum force was decreased by 3,137 N (95% Confidence Interval: 130.0–6,404.5 N). None of the other variables were retained by the model, suggesting that there was no evidence that the explanatory variables were not independently associated to maximum force.

## Discussion

The methodology described herein has been successful in creating fracture patterns on the maxillary fourth premolar teeth that are consistent with those defined by the AVDC under angled compression at forces within the maximum chewing capability of the average domestic dog. There is an inherent variability in the ability of a tooth to resist fracture that would commonly occur during chewing on a treat or toy. The average maximum force sustained by the tested teeth was higher than what has previously been reported for strategically important teeth^2^ (21). The range of data was not unexpected when published information on the fracture performance of human teeth (22) or the fracture behavior of canine teeth of dogs under pulling forces is considered (18). Given the range of breed sizes tested, it is perhaps surprising that the variation was not more significant (23). There was no significant correlation between dog age, dog weight or impact angle and the maximum force required for a tooth to fracture. All three variables did show a positive but non-significant relationship with maximum force. This may be a result of the low number of samples or may indicate that the slope truly is not different to 0 for any variables.

**Figure 7:**
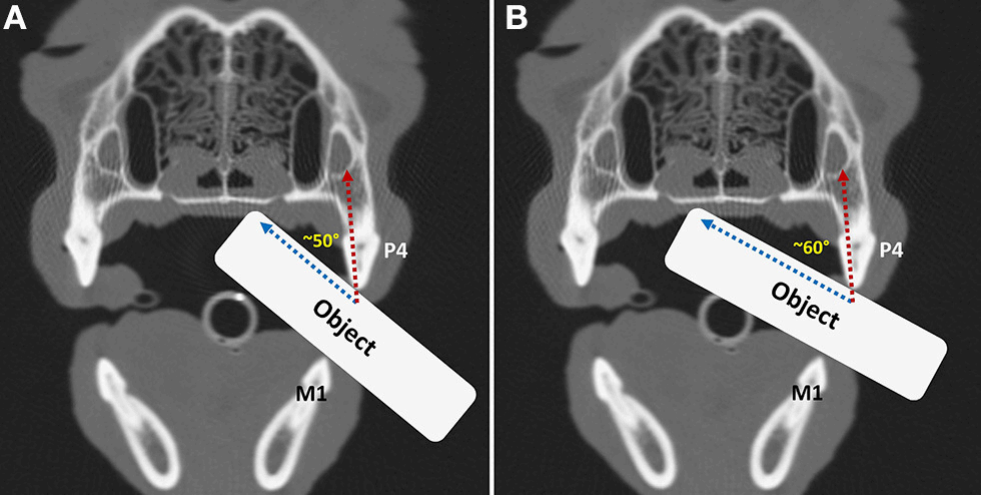
Transverse computed tomography images in bone algorithm obtained at the level of the carnassial teeth in a dog. An object has been positioned from the side in between the left maxillary fourth premolar (P4) and mandibular first molar (M1) teeth prior to **(A)** and beyond the midline of the hard palate **(B)**, demonstrating that the impact angle with the maxillary fourth premolar tooth will change depending on how far the object is inserted into the mouth.

A custom-built aluminum device was used to hold each pot at an angle of 60° with respect to the ground. This angle was chosen after a series of experiments at similar impact angles resulted in fracture patterns that are consistent with those defined by AVDC. It differs from the angle previously used for testing canine teeth (21), but it was hoped that it approximates the vector created when the maxillary fourth premolar tooth occludes against the mandibular first molar tooth with an object in between them, whose intraoral end would meet the hard palate. The angle at which a force is placed on a maxillary fourth premolar tooth likely varies, depending on the shape, surface and size of the treat or toy, the material that it is fabricated with, the physical characteristics of the tooth, and the dog's willingness and ability to chew (Figure 7).

The periodontal ligament of a tooth with healthy periodontium in a living dog likely serves as a shock absorber, but this effect may have been lost due to sample processing in the present study. Detrimental effects of freezing the samples prior to testing cannot be ascertained at this time but may be negligible. When evaluating the effects of freeze-thaw cycles in bone, the effects were found to vary depending on the type of bone assessed, but in most cases were not measureable. If degradation did occur, the effect of freezing on the mechanical properties was smaller than the natural variation of those properties across a sample before freezing (24). A similar study of dental pulp showed that storage in transportation solution for 24 h had no significant negative effects on the histological or mechanical properties of the pulp tissue extracted from the cryopreserved intact teeth (25). When evaluating the effects of freezing on the periodontal ligament, force/displacement curves generated to evaluate the behavior of the periodontal ligament in autopsy specimens were comparable with those previously derived by *in vivo* measurements (26).

Sources of variation may also be due to the history of the dog. Although no visible fracture damage existed on the sampled maxillary fourth premolar teeth, it is possible that microscopic cracks were present in either the enamel or dentin that potentially contributed to an overall reduction in stress resilience (27). The theory of abfraction sustains that tooth flexure in the cervical area is caused by occlusal compressive forces and tensile stresses. These forces result in microfractures of the hydroxyapatite crystals of the enamel and dentin which further fatigue and deform the tooth structure. The thin structure of the enamel and the low packing density of the Hunter-Schreger bands (HSB) at the cervical area in human teeth may contribute to the development of these lesions. Studies have explored the association between occlusal stress and cervical wear by employing finite elemental analysis or photoelastic methods. However, the few clinical studies available were not able to confirm a positive association between occlusal loading and abfraction lesions (27).

The exact geometry of each tooth as well as the thickness and mineralization of the enamel and dentin likely contributed to resistance to fracture. When the raw data trace files from the compression testing are reviewed, small drops in force can periodically be noted along the trace. The minor drops were likely due to the initiation of cracks or fissures in a section of the tooth structure as the stress concentration built. A recent report describes a compression-mediated toughening mechanism owing to the microscopic arrangement of mineral crystals. “On-axis” mineral crystals, associated with collagen, are under compressive stress as a result of strong mineral/collagen interactions. “Off-axis” mineral crystals, not directly associated with collagen, appear to experience far less stress (28). Given the nature of teeth that are inherently designed to resist crack propagation, the force continues to build to catastrophic failure, with the final yield point being defined by the cumulative effects of existing defects, tooth geometry, and quality of the enamel and dentin. The variation may simply reflect the reality of maxillary fourth premolar teeth in dogs. While it might be possible to define inclusion criteria that reduce the range of the data (single dog breed, mono-disperse age range, harmonized feeding regimen, etc.), it was felt that this would detract from the overall relevance of the data because such idealized teeth do not exist in the average dog population.

For the test described in this study, a rigid probe was used to generate fracture patterns that are consistent with those defined by the AVDC in maxillary fourth premolar teeth. Based on the data obtained, a similarly rigid chew material that fails to yield below 1,281 N would be considered to be a risk of fracturing a maxillary fourth premolar tooth. This force is substantially higher than the mean forces required to fracture canine teeth in dogs, which were between 494 and 630 N depending on the crown height to diameter ratio (21), and a fracture force of approximately 890 N has previously been suggested for maxillary fourth premolar teeth.^2^ Crown height to diameter ratio in the present study was significantly associated with fracture force (i.e., a decreased ratio increases tooth fracture resistance), thus confirming what has previously been found for canine teeth (21). However, maxillary fourth premolar teeth in dogs have an inherently lower crown height to diameter ratio compared with canine teeth, which explains the higher forces needed for them to fracture, and three roots rather than one for forces to be distributed along. Indeed, the mean fracture point and all points below have already been shown to be well within the chewing capability of the average dog, and the maximum theoretical bite force of dogs exceeds even the more resistant tooth within this test series (16–18). Domestic dogs likely do not choose to deploy maximum bite forces on a regular basis, and it is likely only during times of high excitement, stimulation of threat or competition that significantly higher than average forces would be exerted as shown by the great variation found in studies that evaluated bite forces in awake dogs (16–19).

A degree of protection from extreme textures would be significant in reducing the risk of chewing-related tooth fracture. This also becomes important in endodontically treated teeth. Ninety percent of A fibers in dental pulp tissue of human teeth are A-delta fibers, which are mainly located at the pulp-dentin border in the coronal portion of the pulp and concentrated in the pulp horns (29). These A fibers transmit signals directly to the thalamus, generating a fast, sharp pain that is easily localized. This can be viewed as a protective mechanism of a tooth in response to an insult. In partial or complete absence of vital pulp tissue the tooth will be rendered less able to detect a noxious stimulus (29). The effects of crown height to diameter ratio, finite element analysis of strategically important teeth, and the mechanical properties of chewing objects currently available in the pet food market should be further tested prior to establishing industry standards for the design of edible and non-edible treats and toys.

The study reported herein describes a methodology for assessing the forces involved in the fracture of sampled maxillary fourth premolar teeth in domestic dogs. Factors that could have impacted the outcome include estimation of dog age and weight, possibility of microscopic tooth weakening prior to sample testing, alteration of the mechanical properties of hard and soft tissues of the tooth and surrounding alveolar bone during sample harvesting, storage and processing, the use of photographs rather than instrumental exploration for fracture type assessment. In addition, the study is limited by the use of point force application and testing to load to failure rather than using a 4-point bending model and cyclic loading to failure. A 4-point bending model would employ forces on the cusps of maxillary fourth premolar teeth to provide downward force vectors on the outside of the object while the cusps of mandibular first molar teeth would provide an upward force vector on the inside of the object. This would mimick the biting down on an object with the upper and lower dental arches at the same time. Furthermore, the study characterized when and how maxillary fourth premolar teeth fail under compressive forces using an incompressible object. The hard indentor could have influenced the fracture modes and forces. If an object were used that also deforms, a computational model (i.e., finite element modeling) would need to be employed to determine how the forces were distributed across the two bodies (specimen and indentor).

Nevertheless, the mean maximum force sustained by the tested teeth prior to fracture was within the maximum chewing capability of the average dog. Dogs routinely exposed to hard treats and toys that do not yield significantly below this point might be at increased risk of fracture of maxillary fourth premolar teeth as a result of overexertion during chewing. Further studies using a larger number of teeth, assessing the influence of the various impact angles and cyclic forces, evaluating the hardness and designs of several treats and toys, and using finite element analysis on maxillary and mandibular carnassial teeth to study 4-point bending forces are warranted to develop a model that predicts fracture modes for a given chewing cycle and object.

## Author Contributions

MS-R: conception and design of the work; sample harvesting; interpretation of data for the work; and drafting of the work. ME: conception and design of the work; and drafting of the work and revising it critically for important intellectual content. MH and SS: conception and design of the work; mechanical testing; and acquisition and interpretation of data for the work. AC-G and LV-M: sample harvesting. DS: performed the statistical analysis. AR: conception and design of the work; sample harvesting; revising it critically for important intellectual content; final approval of the version to be published; and agreement to be accountable for all aspects of the work.

### Conflict of Interest Statement

The authors declare that the research was conducted with funding from Mars Care and Treats Petcare Europe. The reviewer CJS and handling Editor declared their shared affiliation.

## Footnotes

^1^Venturini MAFN. Retrospective study of 3,055 pets referred to Odontovet® (veterinary dental center) during 44 months. Dissertação (Mestre em Medicina Veterinária), Faculdade de Medicina Veterinária e Zootecnia, Universidade de São Paulo 2006;35-37.

^2^Duke A. Forces involved in upper fourth premolar fracture in dogs. Proceedings of the 12th Annual Veterinary Dental Forum, 1998;175.

^3^Correa HL. Fracture strength analyses of canine teeth restored biologically: *in vitro* study. Dissertação (Mestre em Medicina Veterinária). Faculdade de Medicina Veterinária e Zootecnia, Universidade de São Paulo, 2003;1-77.

^4^Carolina Biological Supply Company, Burlington, NC.

^5^Lang Dental, Wheeling, IL.

^6^Instron, Norwood, MA.

^7^National Institutes of Health, Bethesda, MD.

^8^http://www.avdc.org/Nomenclature/Nomen-Teeth.html#fracture
